# Monitoring creatine and phosphocreatine by ^13^C MR spectroscopic imaging during and after ^13^C4 creatine loading: a feasibility study

**DOI:** 10.1007/s00726-016-2294-0

**Published:** 2016-07-11

**Authors:** Barbara H. Janssen, Saskia Lassche, Maria T. Hopman, Ron A. Wevers, Baziel G. M. van Engelen, Arend Heerschap

**Affiliations:** 1 Department of Radiology, Radboud University Medical Center, Nijmegen, The Netherlands; 2 Department of Neurology, Radboud University Medical Center, Nijmegen, The Netherlands; 3 Department of Physiology, Radboud University Medical Center, Nijmegen, The Netherlands; 4 Department of Laboratory Medicine, Radboud University Medical Center, Nijmegen, The Netherlands

**Keywords:** Creatine, MR spectroscopy, ^13^C, Human muscle

## Abstract

Creatine (Cr) supplementation to enhance muscle performance shows variable responses among individuals and different muscles. Direct monitoring of the supplied Cr in muscles would address these differences. In this feasibility study, we introduce in vivo 3D ^13^C MR spectroscopic imaging (MRSI) of the leg with oral ingestion of ^13^C4–creatine to observe simultaneously Cr and phosphocreatine (PCr) for assessing Cr uptake, turnover, and the ratio PCr over total Cr (TCr) in individual muscles. ^13^C MRSI was performed of five muscles in the posterior thigh in seven subjects (two males and two females of ~20 years, one 82-year-old male, and two neuromuscular patients) with a ^1^H/^13^C coil in a 3T MR system before, during and after intake of 15 % ^13^C4-enriched Cr. Subjects ingested 20 g Cr/day for 4 days in four 5 g doses at equal time intervals. The PCr/TCr did not vary significantly during supplementation and was similar for all subjects and investigated muscles (average 0.71 ± 0.07), except for the adductor magnus (0.64 ± 0.03). The average Cr turnover rate, assessed in male muscles, was 2.1 ± 0.7 %/day. The linear uptake rates of Cr were variable between muscles, although not significantly different. This assessment was possible in all investigated muscles of young male volunteers, but less so in muscles of the other subjects due to lower signal-to-noise ratio. Improvements for future studies are discussed. In vivo ^13^C MRSI after ^13^C–Cr ingestion is demonstrated for longitudinal studies of Cr uptake, turnover, and PCr/TCr ratios of individual muscles in one exam.

## Introduction

Creatine (Cr) occurs in fish and meat and is endogenously produced in the liver and kidneys (Guthmiller et al. 1994). It is taken up from blood vessels into tissues with high energy demands such as muscle and brain (Wyss and Kaddurah-Daouk 2000). This uptake is facilitated by a creatine transporter protein (CrT), which transports the compound together with sodium into the cell against a Cr concentration gradient (Guerrero-Ontiveros and Wallimann 1998). Inside the cell, Cr is reversibly phosphorylated to phosphocreatine (PCr) in the creatine kinase (CK) reaction. This Cr–PCr system plays a key role in energy metabolism as a temporal energy buffer and by facilitating substrate diffusion (Meyer et al. 1984). A spontaneous reaction irreversibly degrades both Cr and PCr into creatinine, which is excreted via the kidneys (Wallimann et al. 2011; Hoberman et al. 1948). Because of low PCr levels in some dystrophic muscles (Kemp et al. 1993) and potential cellular protective effects (Pulido et al. 1998), Cr supplementation has been proposed as treatment for several neuromuscular disorders (Kley et al. 2013). Cr is also recognized as an effective nutritional supplement in other diseases and in sports (Wyss and Schulze 2002; Gualano et al. 2012; Kley et al. 2013).

However, selection of an effective Cr loading protocol is hampered by a wide variation in responsiveness between individuals (Harris et al. 1992; Greenhaff et al. 1994). This may be due to various factors determining Cr uptake, such as intracellular Cr concentration, hormone status, and fiber type composition (Mesa et al. 2002; Snow and Murphy 2003). Moreover, the contribution of each factor may vary between different muscles and may change with age or due to disease (Coggan et al. 1992; Kreis et al. 1999; Dubowitz and Sewry 2007; McCall and Persky 2007). Therefore, an in vivo method to directly monitor the supplied Cr in individual muscles would be desirable to help to understand and optimize Cr loading.

Magnetic resonance spectroscopy (MRS) has the unique capability to measure Cr and PCr levels in human tissues non-invasively. With proton (^1^H) MRS, the total amount of Cr (TCr) in muscles can be observed, and with phosphorus (^31^P) MRS, it is possible to monitor PCr (De Graaf 2007). Both ^1^H and ^31^P MRS have been used in studies of TCr and PCr levels in skeletal muscle after Cr supplementation and revealed differential Cr increases ranging from about 5 % in the gastrocnemius to about 23 % in the quadriceps femoris (Vandenberghe et al. 1997; Kreis et al. 1999; Smith et al. 1998, 1999). To examine these two compounds individually in one exam are complicated as different hardware is required for each nucleus and quantitative sequential measurements are hampered by the required calibrations (Brault et al. 2007). By carbon (^13^C) MRS, it is possible to observe separate signals for Cr and PCr simultaneously in skeletal muscle spectra, but because of the low natural abundance of ^13^C in the body, this is not a very sensitive method. However, with oral ingestion of ^13^C-4 labeled Cr, the signal-to-noise ratio can be increased. This was used to establish the PCr/TCr ratio and Cr turnover in the calf of a single individual only employing a surface coil adjacent to the calf for localization, mainly acquiring signal from the gastrocnemius (Kan et al. 2006). Multiple spatially resolved MR spectra can be acquired with MR spectroscopic imaging (MRSI) and, therefore, allow the detection of metabolites in individual muscles (Vermathen et al. 2012). Human muscles have not been assessed yet by ^13^C MRSI to monitor compounds labeled with ^13^C spins at thermal equilibrium.

The aim of the work described in this paper was to investigate the performance of three-dimensional ^13^C MRSI at 3T of the posterior thigh during and after the ingestion of ^13^C-4 labeled Cr. In particular, we wanted to know for different muscles what the PCr/TCr ratio is and how feasible it is to measure Cr turnover and uptake, all in one examination. To test this feasibility of the method for clinical research, we examined a variety of seven volunteers representing potentially different leg muscle conditions, including two patients suffering from facioscapulohumeral muscular dystrophy (FSHD).

## Materials and methods

### Subjects

For this feasibility study, we included four healthy young volunteers (YH), one 82-year-old male (OH), and two genetically confirmed FSHD patients. We made sure that participants had no MR contraindication (pacemaker, metal implants or claustrophobia). None of the subjects performed sports at a professional level, were vegetarian, or were taking Cr as supplement.

Participants’ demographics are listed in Table 1. Ethical approval was obtained through the institutional review board, and written informed consent was obtained from all participants.

**Table 1:** Participants demographics

	Age (years)	BMI (kg/m^2^)	Weight (kg)	Gender
Young healthy #1	23	20.94	57	Female
Young healthy #2	21	20.62	58	Male
Young healthy #3	21	22.06	63	Female
Young healthy #4	21	21.90	85	Male
Old healthy	82	30.67	95	Male
FSHD #1	72	33.46	90	Female
FSHD #2	41	24.68	73	Male

Fat fractions were similar between young healthy man and women (1.2 ± 0.3 % vs. 2.8 ± 0.8, *p* = 0.08 two-tailed *t* test)

### Study design

Participants ingested 20 g Cr per day for 4 days in four 5 g doses (15 % ^13^C enriched at the guanidino carbon, ^13^C-4–Cr) at equally spaced intervals through the day. They were instructed to dilute the Cr in a glass of lukewarm water and to drink it along with 250 ml of standard carbohydrate solution to augment skeletal muscle Cr accumulation (Green et al. 1996).

After the first MR measurement on day 0, the participant was provided with 16 portions of the ^13^C-enriched Cr, and was instructed to start the Cr supplementation the next day.

The full study design consisted of 12 MR measurements at day 0, 1, 2, 3, 4, 5, 7, 12 ± 1, 14 ± 1, 19 ± 2, 41 ± 3, 78 ± 3, and 105 ± 3. Day 0 was the baseline measurement, days 1–4 were during Cr supplementation, and the remaining measurements were after Cr supplementation. The measurement protocol was reduced to six MR exams for the older and FSHD subjects; one before Cr supplementation (day 0), two during (days 2 and 5), and three after (days 19, 41, and 78) Cr supplementation.

Blood and urine samples were taken on days 0, 3, and 7 to monitor kidney function during Cr supplementation. Venous blood was drawn directly into a K_2_-EDTA vacuum tube. Tubes were centrifuged at 3000 revolutions per minute for 10 min, and subsequently, the plasma was separated from the packed red blood cells and was stored at −80 °C until analysis. Cr and ^13^C-4–Cr concentrations were determined with mass spectroscopy.

### MR protocol

MR exams were performed on a clinical 3Tesla MRI system (Tim Trio, Siemens, Erlangen, Germany) with a ^1^H birdcage volume coil (diameter 25 cm) and a circularly polarized ^13^C half-volume coil (13 × 15 cm) inserted into the ^1^H coil. A fish oil capsule served as a matching landmark between the MR exams and was positioned at one-third the distance between the anterior superior iliac spine and the patella. Subjects were placed feet first supine inside the magnet bore, and the coil was placed around the right upper leg. After scout imaging, the protocol consisted of T2 multi-spin-echo imaging (T2-MRI; Kan et al. 2009) (field of view 175 × 175 mm, voxel size 0.68 × 0.68 mm repetition time (TR) 3000 ms, 16 equally spaces echo times (TE) 7.7–123.2 ms, 8 slices, slice thickness/gap 6/9 mm).

Followed by 3D, ^13^C MRSI with an adiabatic BIR45 excitation pulse centered at 157 ppm, which is between the resonance frequencies of Cr and PCr. The FOV was 200 × 200 × 400 mm, and the matrix size was 10 × 10 × 8, resulting in nominal voxels of 20 × 20 × 50 mm. Furthermore, Hamming-weighted k-space acquisition was applied with TR 1000 ms, bandwidth 10 kHz, vector size 1024, and 30 averages with WALTZ16 proton decoupling during acquisition.

In one subject, a T_1_ measurement was performed at day 10, by acquiring ^13^C free induction decays (with adiabatic excitation and 150 averages) at different repetition times (TR = 0.75, 1, 2, 3, and 8 s).

**Fig. 1:**
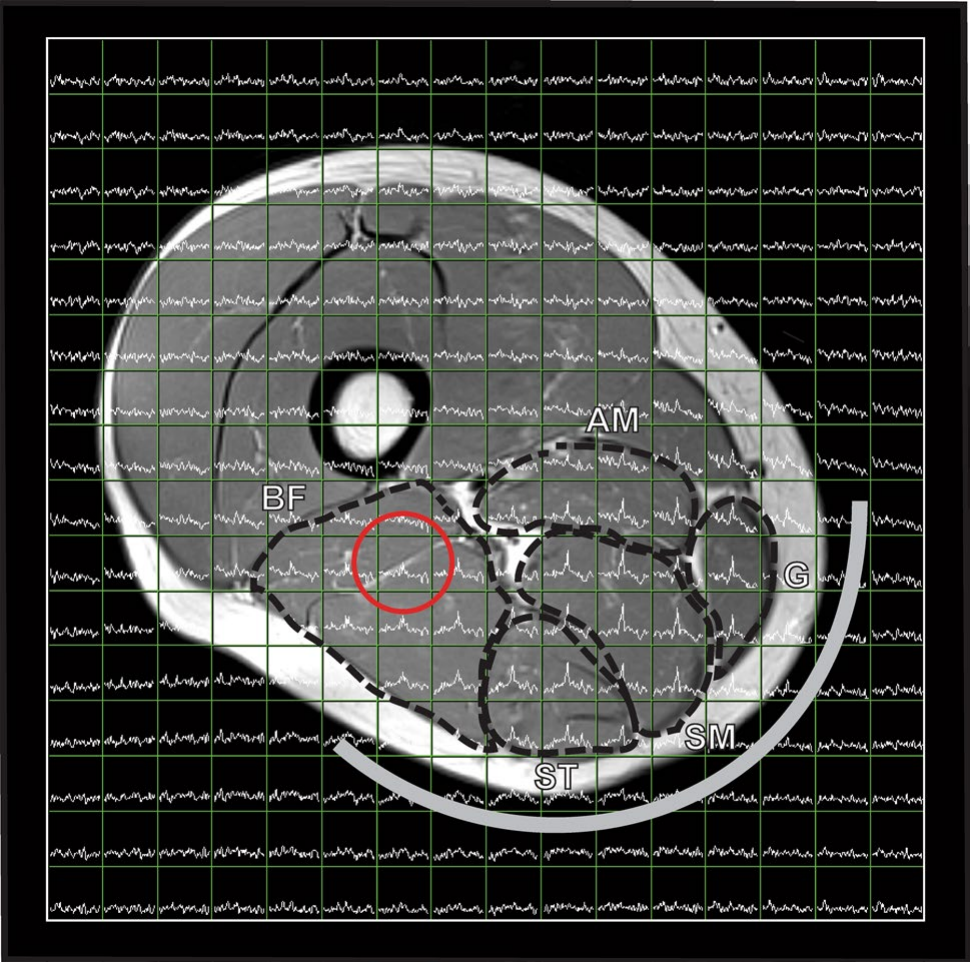
^13^C MRSI grid with voxels overlaid on T2-weighted MR image of the right thigh of a healthy young male after 4 days of creatine supplementation. Because of the *k* space sampling, the true voxel shape is more a sphere, which is approached in the image by a* red circle*. The ^13^C half-volume coil, positioned adjacent to the hamstring muscles, is indicated. The spectra are zoomed on the creatine signals. The muscles that are in the field of view of the coil include the biceps femoris (BF), semi-tendinosus (ST), semi-membranosus (SM), gracilis (G), and adductor magnus (AM), as delineated in the image (color figure online)

Because of the ^13^C coil sensitivity profile only spectra from the posterior thigh muscles, more specifically, the biceps femoris (BF), semimembranosus (SM), semitendinosus (ST), adductor magnus (AM), and the gracilis (G) were collected (Fig. 1). The entire MR examination took about 1 h, including subject placement. All MRSI data sets were saved as raw files for off line processing.

### Data analyses

Muscle specific fat-fractions were determined by fitting the signal of the multi-spin-echo images to a biexponential function with fixed relaxation times for fat and water as described previously (Kan et al. 2009), classifying severe fatty infiltration as a fat fraction above 80 % and minor as below 20 %.

For the ^13^C MRSI data, the matrix dimension was zero-filled to 16 × 16 × 8. A spatial 100 % Hamming filter was applied to the ^13^C MRSI k-space data before voxel selection. From every slice in the field of view of the ^13^C coil, one voxel was selected per muscle using the program 3DiCSI (Zhao et al. 2005). To position the voxel in the center of the muscle, grid-shifting was applied. Only voxels were selected that were completely lying within one muscle, resulting in three voxels per muscle on average. The corresponding free induction decays (FID) were exported to jMRUI 4.0 for spectral fitting (Naressi et al. 2001).

#### Spectral fitting

Voxels from the same muscle were summed, subsequently, unwanted lipid resonances were removed from the ^13^C spectra with an HLSVD filter (Pijnappel et al. 1992), and the spectra were apodized by 10 Hz. From unlocalized high SNR MR spectra, a line width ratio of 0.81 and a frequency difference of 30 Hz between Cr (157.5 ppm) and PCr (156.6 ppm) were obtained as prior knowledge that was used in the fit of the signals to Gaussian line shapes using AMARES software (Vanhamme et al. 1997). With the Cr and PCr pools in equilibrium (Brault and Terjung 2003), the summed signal intensities of Cr and PCr served as TCr. Spectral quality was assessed by comparing the TCr signal intensity to the standard deviation (SD) of the noise (defined as SD of the last 10 % of the FID). Only spectra in which the TCr signal intensity was larger than the SD of the noise were included for further analysis. In addition, we excluded spectra in which the fitted line width of the ^13^C creatine signals was more than 50 Hz.

#### Creatine uptake

TCr amplitudes of individual muscles at days 0 till 5 were fitted to a linear function (Eq. 1) where TCr (*t*) is the ^13^C signal intensity at measurement day *t*. This was only calculated for the muscles that had at least 3 measurement days with spectra that passed the quality criteria (see above). The fitted slope gives the Cr uptake rate (*k*_up_) and the intercept the initial TCr value (TCr_0_), both in arbitrary units (a.u.). The final uptake rate is presented as *k*_up_/TCr_0_ to give a value expressed in percentage per day. The error of the fit was treated as the standard deviation.

1 $${\text{TCr}}(t) = k_{\text{up}} t + {\text{TCr}}_{0}$$

#### Creatine turnover

TCr signal intensities from day 5–105 were used to calculate the turnover rate. Cr breakdown is assumed to follow first order kinetics (Walker 1979), so TCr signal intensities were fitted to a mono-exponential function (Eq. 2), under the assumptions described in (Kan et al. 2006). The maximal ^13^C TCr signal intensity is TCr_max_, and *k*_br_ is the breakdown rate (% per day). The error of the fit was treated as the standard deviation. All curve fitting was done using Prism 5.0 (GraphPad Software, San Diego, CA, USA).

2 $$\frac{{{\text{TCr}}(t)}}{{{\text{TCr}}_{0} }} = \frac{{({\text{TCr}}_{\hbox{max} } - {\text{TCr}}_{0} )e^{{ - k_{\text{br}} t}} }}{{{\text{TCr}}_{0} }} + 1$$

#### PCr/TCr ratio

The PCr/TCr ratio was determined per muscle over time for every measurement point unless the Cr and PCr signals could not be resolved properly (i.e., one or both signals fitted with amplitude 0). To compare the PCr/TCr ratios between individual muscles, the PCr/TCr ratios were averaged over all these measurement points.

#### T_1_ measurement

The Cr and PCr signal intensities were fitted as a function of the repetition time according to Eq. 3, giving a value for the T_1_ relaxation time in ms.

3 $$\frac{{M_{z} ({\text{TR}})}}{{M_{0} }} = 1 - e^{{ - {\text{TR}}/T_{1} }}$$

### Statistics

A one-sided paired *t* test was used to compare blood plasma concentrations to day 0, and to assess differences between male and female muscles. Errors are given as standard deviation (SD) unless presented otherwise. Muscle specific effects of Cr uptake and PCr/TCr ratios were assessed by a one-way ANOVA. All statistical analysis were done using Prism 5.0 (GraphPad Software, San Diego, CA, USA), and statistical significance was set at *p* < 0.05.

## Results

### General observations

None of the subjects reported side effects of the creatine supplementation. Blood analysis of the creatine content and ^13^C enrichment indicated that all subjects adhered to the prescribed intake of the supplementation (Table 2). At day 3, these values were comparable for young males and females, i.e., average Cr content was 929 and 711 μM, respectively, and average ^13^C enrichment 22 and 20 %, respectively. Two days after the supplementation period the plasma creatine levels had returned to normal values, but the ^13^C enrichment was still increased.

**Table 2:** Plasma creatine concentrations and percentage ^13^C-4–Cr (mean ± SD)

	Day 0	Day 3	Day 7
Plasma creatine (µM)	38 ± 23	668 ± 432**	41 ± 6
%^13^C	6.0 ± 0.6	19 ± 6.1**	10 ± 0.5**

Those at day 3 and day 7 are compared with day 0 by means of a one-sided paired *t* test

** *p* < 0.01

**Fig. 2:**
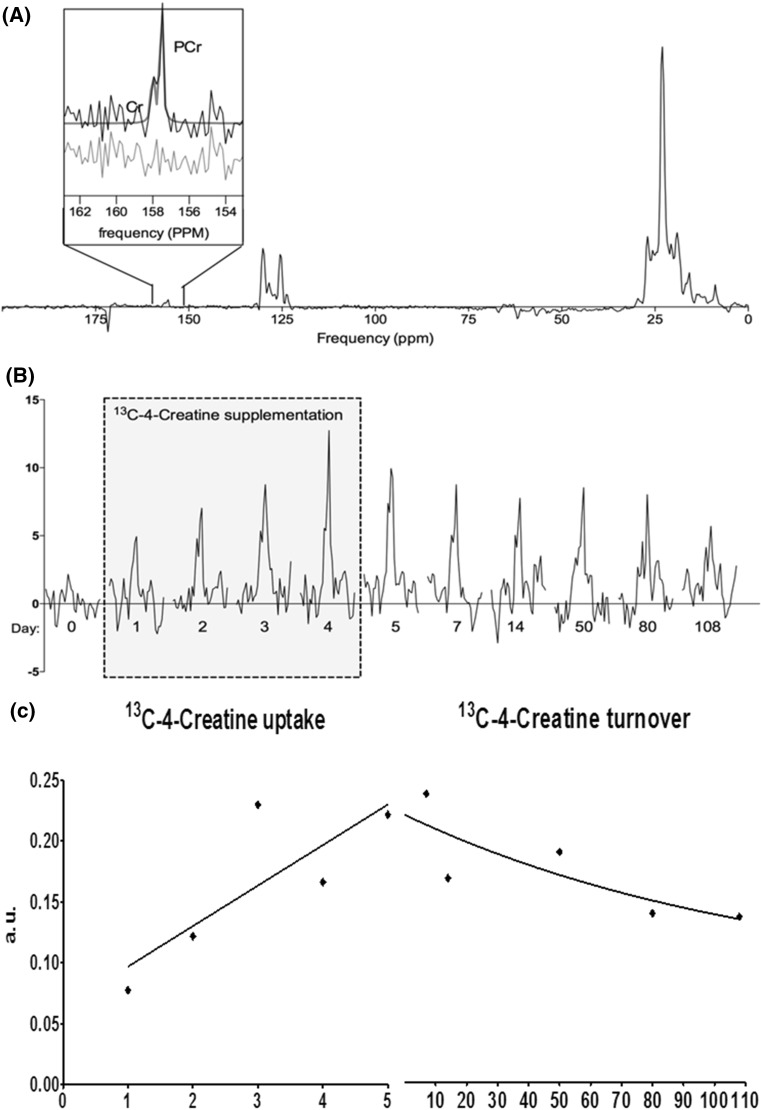
^13^C MR spectra before, during and after creatine supplementation in the ST muscle of a healthy young male subject. **a** Full ^13^C MR spectrum of this muscles at day 7, 3 days after the creatine supplementation period. Cr and PCr resonate at 157.5 and 156.6 ppm, respectively. Even though creatine is 15 % ^13^C enriched the signal intensity is still much smaller than that of natural abundance ^13^C lipid resonances. The* inset* shows the fit of the PCr and Cr resonances with the residual below. **b** Starting with day 0 a clear increase in signal intensity is observed during the 4 days of 13C-4–Cr supplementation followed by a slower decrease; the signal is still elevated up to day 108. **c** The uptake rate is fitted with a linear function over the first 5 days, and the breakdown rate was fitted by a mono-exponential decay. Note that for the uptake part, the integral of the creatine signals at day 0 is not included as the fit of these signals did not pass the quality criteria

During the ingestion of 20 g/day 15 % enriched ^13^C-4–Cr for 4 days, the ^13^C signals for PCr and Cr increased in muscles within the sensitive profile of the ^13^C/^1^H coil (Figs. 1, 2b). The separate signals of PCr and Cr were integrated by fitting, using the prior knowledge described above (Fig. 2a, inset), for further analyses.

### Creatine uptake rate

**Fig. 3:**
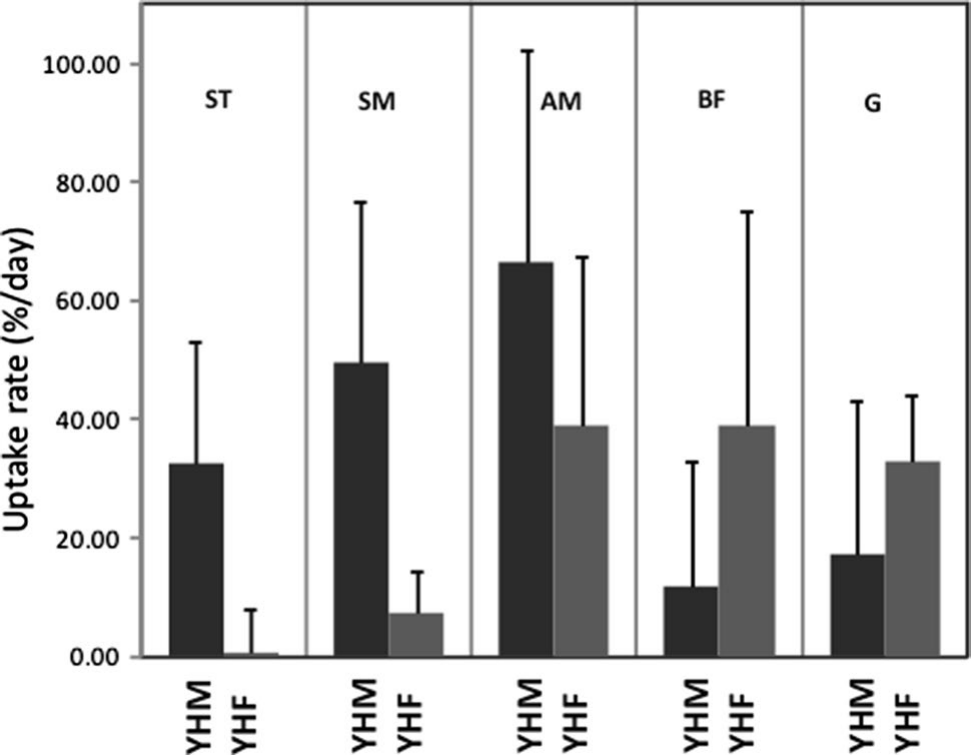
Muscle specific creatine uptake rates (% per day) in four volunteers: two young healthy males (YHM; *black bars*) and two young healthy females (YHF; *gray bars*), from *left* to *right*: semi-tendinosus (ST), semi-membranosus (SM), adductor magnus (AM), biceps femoris (BF), and gracillis (G). Each uptake rate is the average of two muscles, except for the ST, BF, and G in YHF, for which the *bars* represent the uptake rate in one muscle. For the latter muscles, the *error* of the linear fit (see Fig. 2c) is given as SD. In the presented SD of the other muscles, these fitting *errors* have been considered

The summed intensities of the ^13^C signal of Cr and PCr determined at day 0 till day 5 were fitted to a linear function (Fig. 2c) to yield a subject and muscle specific Cr uptake rate. Following the spectral quality criteria, this analysis could be performed in 17 out of the 20 investigated muscles of the young healthy volunteers. In males, a significant Cr uptake rate was observed in the SM, ST, and AM muscles with less in the other two muscles (Fig. 3). In females, we could observe Cr uptake in all investigated muscles, except that the G muscle of one female was too thin to place a voxel solely in that muscle. The data of the BF and ST muscles of this female did not pass the quality criteria to obtain a Cr uptake rate mainly because of low signal-to-noise ratio in some spectra. Taking all remaining muscles together the mean uptake rate in females was lower than in males although not significant (17 ± 26 vs. 36 ± 22 %/day with SD; *p* = 0.25).

To test the feasibility of the ^13^C MRSI assessment of muscular creatine uptake in more fragile persons, i.e., a 82-year-old male and two FSHD patients, it was necessary to decrease the number of exams during the supplementation and thereafter. The creatine supplementation was well tolerated by all participants, and no side effects were reported.

The female FSHD patient (FSHD#1) showed fatty infiltration in the AM and SM muscles, and the male FSHD patient (FSHD#2) showed severe fatty infiltration in the SM muscle, and minor fatty infiltration in the ST and BF muscles. Following the spectral and fitting quality criteria, creatine uptake rates could be determined for FSHD#1 in the ST (48 ± 101 %/day), AM (27 ± 5 %/day), and the BF (9 ± 12 %/day), and for FSHD#2 in the BF (77 ± 184 %/day). For the 82-year-old male, we observed Cr uptake in four of the five investigated muscles of which three passed the quality criteria. The SM had an uptake rate of 8 ± 15 %/day, the BF 9 ± 28 %/day, and the G 24 ± 8 %/day.

### Creatine turnover

**Fig. 4:**
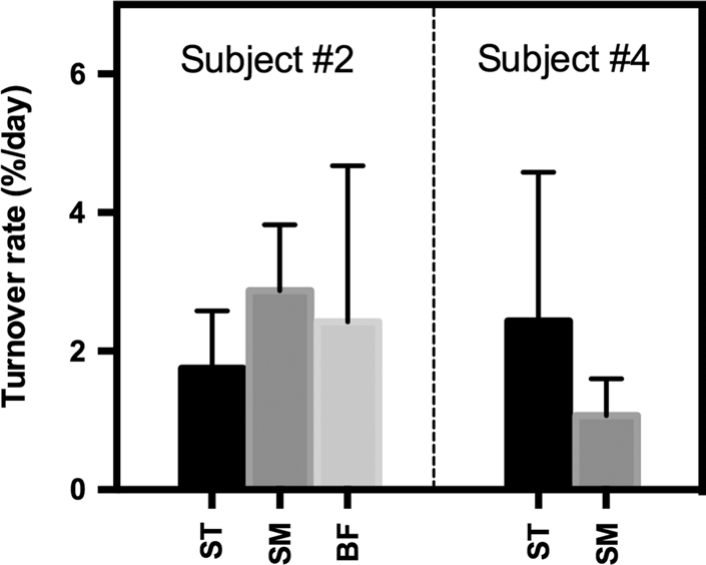
Muscle specific Cr turnover rates (% per day) in young male volunteers. Only muscles in which the turnover signals were found to follow a mono-exponential decay were included. Turnover rate could not be determined if muscles showed relatively low Cr uptake. *Bars* indicate the mean uptake rate with standard error of the mean

The Cr turnover was determined by fitting the TCr signal intensities from day 5–105 after start of supplementation, to a mono-exponential decay function (Fig. 2c). Because all Cr curves will fit to this function, even when no Cr uptake had taken place, we added a fit quality criterion that *R*^2^ has to be larger than 0.5 to ensure that the fitted values indeed follow a mono-exponential decay. Only data of three muscles of male subject #2 and two muscles in the other male fulfilled this criterion. No turnover rates fulfilling the fit quality criteria could be determined for the female volunteers. For the muscles in which the Cr turnover could be determined, the rates were comparable with an average of 2.1 ± 0.7 % per day (Fig. 4).

### T_1_ relaxation times of ^13^C-4 spins

To verify that the pulse repetition time for MRSI acquisition did not cause signal saturation, we measured the T_1_ relaxation times of the ^13^C spins in the hamstring muscle of one healthy young male subject by analyzing the ^13^C-4 signals of Cr and PCr as a function of the repetition time. The T_1_ for Cr was 341 ± 36 ms and for PCr 413 ± 49 ms. This implies that with a 45° excitation pulse and a TR of 1000 ms for the ^13^C MRSI acquisition, there will be little spin saturation.

### PCr/TCr ratio

**Fig. 5:**
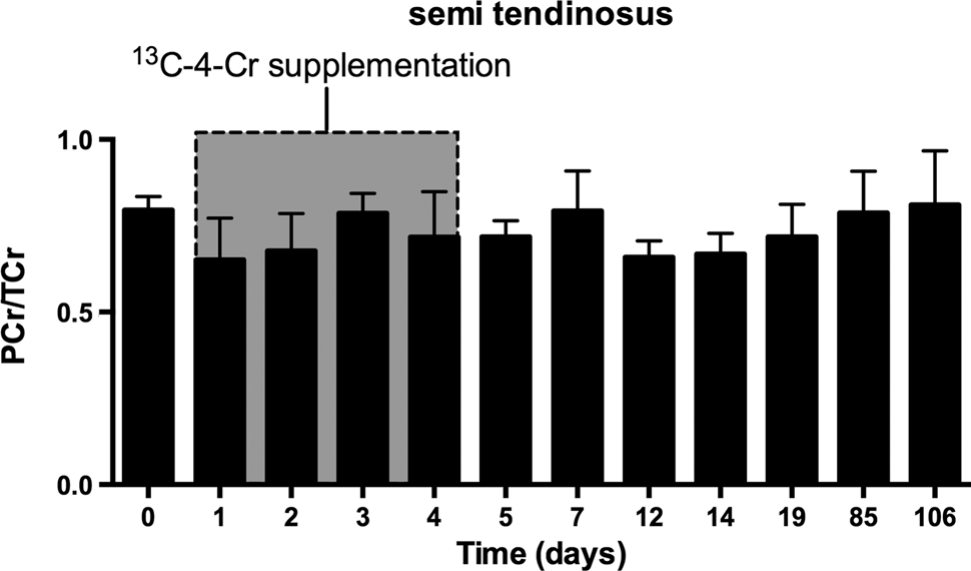
PCr/TCr ratios of the semi-tendinosus muscle before, during and after Cr supplementation of the four young healthy volunteers. PCr/TCr ratios were determined from ^13^C spectra acquired and analyzed as described in the materials and methods section. *Bars* indicate the mean values, and the *error bars* indicate the standard deviation

To investigate if creatine supplementation would change the PCr/TCr ratio, we determined this ratio for every time point during and after the supplementation. For none of the examined muscles in the healthy young volunteers, we observed a significant change in the PCr/TCr ratio during this period. An example is shown in Fig. 5 for this ratio in the ST muscle, which is around 0.75.

**Fig. 6:**
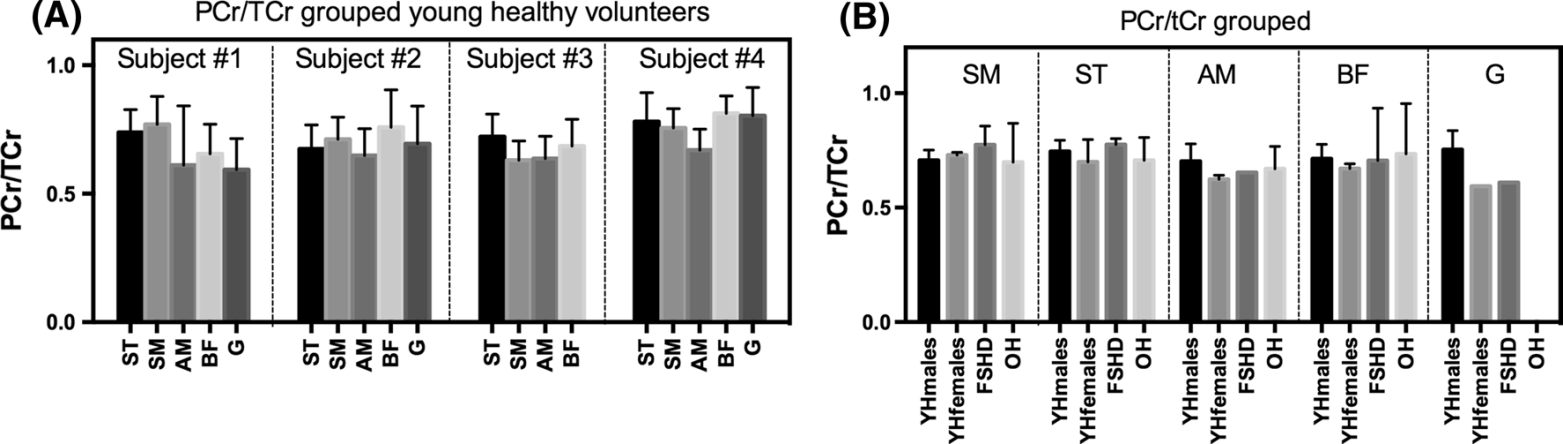
PCr/TCr ratios. **a** Muscle specific ratios in young healthy volunteers (*n* = 4). One-way ANOVA did not reveal statistical significant muscle specific effect. *Bars* indicate the mean PCr/TCr per volunteer and the *error bars* indicate standard deviation. **b** Grouped data of the PCr/TCr ratio of the young healthy subjects, the FSHD patients, and the old volunteer. The PCr/TCr ratios of the FSHD and old subjects are within the range found in the young healthy subjects. *YHmales/females* young healthy subjects, *FSHD* FSHD patients, *OH* old healthy subject. *Error bars* indicate standard deviation

To investigate if the PCr/TCr ratio is the same as determined in other studies, we assessed this ratio for each investigated muscle in the young volunteers averaged over all time points (Fig. 6a; Table 3). Testing each muscle against the other revealed that the PCr/TCr of the AM is significantly lower than that of the other muscles (*p* < 0.05), but a one-way ANOVA did not show a muscle specific effect for the PCr/TCr ratio (*p* = 0.32). For all subjects and all muscles, together the average PCr/TCr ratio was 0.71 ± 0.07 (including a small correction for differences in T_1_ between PCr and Cr).

**Table 3:** Average muscular PCr/TCr ratios of the four young volunteers (mean ± SD)

Muscle	Mean
ST	0.75 ± 0.04
SM	0.73 ± 0.03
AM	0.65 ± 0.03
BF	0.75 ± 0.07
G	0.70 ± 0.10

With correction for the T_1_ values of Cr and PCr

The average PCr/TCr ratios of all investigated muscles in the old healthy male and FSHD patients were similar to the values of the young healthy subjects (see Fig. 6b), with T_1_-corrected values of 0.71 ± 0.03 and 0.71 ± 0.07, respectively.

## Discussion

In this feasibility study, we demonstrate that 3D ^13^C MRS imaging with oral ingestion of ^13^C4–creatine can be used to monitor Cr uptake and turnover rates as well as PCr/TCr ratios in individual muscles of the human leg. Previously, we showed with unlocalized ^13^C MRS, and an RF coil next to the gastrocnemius of a male subject that the non-invasive monitoring of Cr and PCr during and after supplementation of ^13^C4-labeled Cr is feasible (Kan et al. 2006). Given the substantial variation in creatine uptake in different muscles, it is relevant to demonstrate that with ^13^C MRS in spectroscopic imaging mode, we can spatially resolve information of ^13^C labeled metabolites in multiple individual muscles in one exam. We applied this technique in a diverse group of subjects, including four young and one old healthy volunteer, and two patients with FSHD. In this way, the supplied Cr, and its immediate cellular product PCr, is assessed instead of the total Cr or PCr pool, as has been done with ^1^H or ^31^P MRS after oral Cr ingestion, e.g., (Kreis et al. 1999; Brault et al. 2007).

It is a common finding in human muscular studies of creatine uptake, assessed with biopsies, that TCr increases more than PCr during the supplementation period, e.g., (Hultman et al. 1996; McKenna et al. 1999). A study combining ^1^H and ^31^P measurements to determine TCr and PCr in the vastus lateralis (where most biopsies are taken from) did not find this (Brault et al. 2007). The authors attribute this discrepancy to higher PCr hydrolysis during the biopsy procedure because of the change in physiological condition of muscles loaded with Cr. Our single subject study using ^13^C MRS also did not find a change in the PCr/TCr ratio of the gastrocnemius during Cr uptake (Kan et al. 2006). In the present study, we assessed this for a few other muscles in the thigh and found no preferential increase in TCr over the supplementation period within the error of the measurements. These findings indicate a general preservation of the overall thermodynamic equilibrium of the creatine kinase reaction in muscle cells at rest, also during Cr loading.

The range of average PCr/TCr ratios of the investigated muscles (from 0.65 to 0.75) is within the range reported in the literature for this ratio from 0.6 to 0.8 (Casey et al. 1996; Mesa et al. 2002) and comparable with our previous unlocalized study of the gastrocnemius (Kan et al. 2006). The PCr/TCr ratio is similar for all the investigated muscles in the young volunteers with a somewhat lower value for the adductor magnus, which may be due to a different fiber type distribution (Johnson et al. 1973; Mesa et al. 2002).

Interestingly, the average PCr/TCr ratios in the older subject and FSHD patients were not different from the young volunteers indicating that energy balance and CK activity in the muscle of these subjects result in a similar thermodynamic equilibrium of the reaction at rest. In principle, muscle specific PCr/TCr ratios can also be determined non-invasively with ^1^H and ^31^P MRS recordings of the same muscle, but this has not yet been reported as such.

The average muscular Cr turnover rate of 2.1 ± 0.7 % per day is in agreement with the results of our previous ^13^C MRS study of a single subject and with the reported non-enzymatic breakdown rates of creatine to creatinine (Kan et al. 2006; Walker 1979; Wyss and Kaddurah-Daouk 2000).

In particular, for muscles with relatively high SNR spectra (SM and AM muscles of young volunteers), the data points indicate an apparent linear uptake of labeled Cr during the 4 days supplementation. This is in agreement with previous supplementation studies and has been associated with near-saturation of the Cr transporters (Brault et al. 2007), which would suggest that uptake rates reflect creatine transporter capacity of muscles.

Cr uptake rates were variable among the five thigh muscles and subjects and varied between 0 and about 80 % per day over all subjects and muscles. It is not possible that the lack of Cr uptake in some muscles is caused by subjects not taking ^13^C-labeled Cr as the blood plasma samples analyzed before and during the Cr supplementation period showed similar increase in Cr and ^13^C-4–Cr concentration for all subjects during the supplementation period proving that they did ingest the provided ^13^C–Cr.

Differences in Cr response have been observed in rat hind limb muscles, with significant uptake in the gastrocnemius muscle but no response in the soleus muscle, an effect attributed to a higher contribution of type II fibers in the gastrocnemius (McMillen et al. 2001). In humans, the increases in (relative) Cr or PCr level after supplementation appear to differ among leg muscles with reported values from about 4 % for the gastrocnemius (Vandenberghe et al. 1997) to about 20 % for quadriceps muscles (Kreis et al. 1999; Smith et al. 1999). The origin of these differences is unknown; in studies addressing this issue, the proposed ^13^C MRSI method to assess uptake rates could be helpful.

Based on the reported values for Cr turnover rates, we estimate that to keep up with spontaneous constant Cr breakdown, a ^13^C signal increase in muscle spectra of 10–30 %/day is expected during the supplementation. This implies that higher uptake rates are reflecting an increase of Cr levels in the muscles. It also suggests that for muscles with an uptake rate below about 10 %/day in this study, the SNR and/or the reproducibility of the measurement and/or fitting procedure of the Cr signal was inadequate.

For this ^13^C MRSI study, we increased the field strength from 1.5T to 3T and the ^13^C enrichment from 10 to 15 %, compared with our previous ^13^C MRS study (Kan et al. 2006). The higher sensitivity allowed us to study ^13^C uptake and metabolism in individual muscles of young male subjects with sufficient SNR. However, this was less so in the females and older person and in FSHD patients, in which the quality filter only allowed to assess an uptake rate in four out of ten muscles. In the latter cases, this could be due to less Cr uptake or a lower muscular fraction. In a ^31^P MRS study on FSHD patients, a preferential decrease of the SNR and the PCr/ATP ratio was observed in fat infiltrated muscles (Kan et al. 2010). These results imply that for future clinical research studies with ^13^C MRSI, the SNR has to be improved, which can be achieved in several ways. First, the relative short T_1_ values of the ^13^C spin systems in Cr and PCr (≈400 ms) at 3T allow for a shorter Tr than the currently used 1000 ms, so that more scans can be acquired in the same amount of time. Second, the voxel size can be enlarged in the length direction of the muscles. Third, improved MR instrumentation can be used, such as array RF coils or higher field strength, which requires substantial investments. Fourth, the ^13^C enrichment of the creatine can be increased, which, however, will increase the cost of the experiment. In addition, calibrations and registrations for better reproducibility, and signal fitting with more restrictive prior knowledge, such as line width limitations, may help.

For a full non-invasive investigation of muscular creatine uptake, it would be important to also know the total amount of creatine during and after the supplementation (Nabuurs et al. 2013). As we already employ a proton RF coil in the current setup, this can be most easily achieved by simultaneous or alternate ^1^H MRS measurements (Kreis et al. 1999).

In summary, we introduce an in vivo ^13^C MRS imaging method to study intake and conversion of ^13^C labeled metabolites in skeletal muscles. We demonstrate that this allows for direct monitoring of supplied ^13^C4-labeled Cr for subject and muscle specific determination of PCr/TCr ratios, and Cr turnover and uptake rates.
